# Some Practical Observations on Small Pox and Vaccination, at Rock Island Prison Barracks, Rock Island, Ill.

**Published:** 1864-06

**Authors:** R. M. Lackey

**Affiliations:** A. A. Surg. U. S. A., M. D.


### SELECTED.

---

### SOME PRACTICAL OBSERVATIONS

### ON SMALL POX AND VACCINATION,

*At Rock Island Prison Barracks, Rock Island, Ills.*

By R. M. LACKEY, A. A. Surg. U. S. A., M. D.

There are few subjects in the medical world that have claimed the attention and study of physicians more than small pox. Every medical writer for centuries past has treated of it, some devoting themselves exclusively to its study, the most valuable results attending their labors. It is, however, a source of regret that, nearly three-quarters of a century after the achievements of Jenner, small pox should still prevail, notwithstanding we have daily evidence of the power of vaccination, when properly employed, to arrest the ravages of this loathsome disease. From its earliest recorded history small pox seems to have prevailed extensively in large armies, the presence of which, in this country during the past three years, I think may be regarded as the exciting cause of its general prevalence, the contagion becoming more powerful by concentration, the predisposing cause being negligence in regard to vaccination, and the large number of persons who are consequently unprotected.

The appearance of the disease here was almost simultaneous with the first arrival of troops for garrison duty, but it did not begin to spread to an alarming extent until a fortnight or more after the arrival of the first lot of prisoners, which was about the 5th of December, 1863. From Jan. 1st, 1864, to about March 1st, it spread rapidly and almost entirely among the prisoners; some days as many as forty new cases occurred. As there had been no buildings erected for hospital purposes outside the prison yard some old dwelling-houses on the island were used as a Pest-Hospital, and men sick with small pox were crowded into these, where they suffered from exposure and insufficient ventilation, until better accommodations were provided. Although there are few who have never been vaccinated, yet the number of bad cases that occur is large; thus out of 558 cases admitted, there were but 44 that had never been vaccinated; yet out of this number 166 had confluent small pox. This may be accounted for by the fact that, of the 514 that had been vaccinated, there were 107 on whom the vaccination had not taken effect, and 59 in whom the matter used was evidently bad, as is shown by the character of the scars; and in 38 cases the variola and vaccine disease occurred simultaneously, making in all 204 that were unprotected. The whole number admitted to small pox hospitals to this date is 1165; of these 335 have died, or one in 3.17. Of confluent cases the deaths have been about 75 per cent. This would show a heavy mortality but for the condition of these men when attacked. Many of them are almost exhausted by other diseases, and a large number die during the first week of the eruption.

In the treatment of small pox several plans and remedies have been employed, and pronounced by some to have the power of aborting the disease. The Saracenia Purpurea, so highly recommended by Dr. Morris, of Halifax, N. S., and others, has been used here to some extent, but its employment has not been followed by the marvellous effects claimed for it; as soon, however, as another supply can be procured, we propose to give it a further trial, and hope to be able to report more favorably.

It has been asserted by some that vaccination, even after the variolous eruption has appeared, modifies the disease, and this course has been recommended; but observations here have not confirmed the truth of this statement. I have carefully observed thirty-eight cases in which the vaccine disease and small pox occurred simultaneously, and there was not a case in which the vaccination seemed to be of any benefit; but in some cases the variolous modified the vaccine poison, making the vesicle smaller than usual, and in others the vaccine vesicle became contaminated with the variolous poison, and ran the same course as the small pox pustules. I see no object, therefore, in vaccinating after the variolous eruption has appeared.

The treatment which seems to be attended with the best results here is, to open the bowels freely at the onset of the disease, and, if there be much nausea, an emetic may be advisable to assist in freely unloading the stomach. Some of the saline laxatives are given to keep the bowels soluble during the course of the disease. For restlessness and wakefulness Dover's Powder in 10 gr. doses at bedtime. After the secondary fever has subsided it is necessary to use all the supporting means at our disposal—quinine, wine, iron, egg-nogg, and nourishing diet. For the throat affection that so frequently occurs from about the sixth to the eighth day, we have been using bromine by inhalation, and with very decidedly beneficial results. Frequently the tongue and throat swell enormously in a few hours, so that the patient can neither speak nor swallow, and suffocation seems imminent. In these cases we have seen the swelling diminish as rapidly as it came, from the use of this remedy. An inhaling apparatus may be extemporized by two tubes placed in the cork-stopper of a wide-mouthed bottle. From the fourteenth to the twenty-first day, pneumonia is most to be feared, and nearly all the cases in which it occurs prove fatal. Treatment is of but little avail, except to palliate as far as possible the sufferings until death relieves the victim. Erysipelas is very prevalent; nearly 25 per cent. of the whole number of patients are in the erysipelas ward. For this we find the local and constitutional use of iodine and bromine, with prompt support, the best treatment. Abscesses and sloughing are very common among anæmic subjects. I have seen the scrotum and penis nearly all slough away before death took place. For the abscesses, when they become extensive and the parts gangrenous, we have found the injection of a weak solution of iodine, after the pus is evacuated, very beneficial.

We have made use of a variety of external local applications—mainly for their soothing effects during the stages of maturition and decline. A very excellent soothing application is olive oil and kreosote, from ten to twenty drops of the latter to the ounce of oil. The oil softens the surface, and the kreosote allays the itching, and, besides, its antiseptic oroperties are of some value. Another local remedy that we have used with great advantage, especially for the eyes, is glycerine. We usually take equal quantities of glycerine and water, and for the eyes the addition of a few grains of tannin to the ounce is beneficial. Where there are extreme dryness and soreness of the mouth, the glycerine mixture is an excellent application, not only in small pox but in other diseases.

Of the 558 cases of which I have taken notes, those between 15 and 20 bear the disease best; no bad cases occurring in those over 40 years of age recover; and no deaths have occurred in those who have a good vaccine mark.

Vaccination is the great disarmer of this terrible destroyer of human life; and the zeal manifested by some physicians in extending its blessings soon after its discovery is well worthy of imitation. As early as 1803 a voyage round the globe was undertaken for the sole purpose of extending vaccination. An account of it, translated from the *Madrid Gazette*, of October 14, 1806, begins as follows: "On Sunday, the 7th of last September, Dr. Francis X. de Balmis, honorary Surgeon of the Royal Chamber, had the honor of kissing the hand of his Majesty on his return from a voyage round the world, undertaken with the sole purpose of carrying to all the Spanish dominions beyond the sea, as well as to those of other nations, the inestimable blessing of vaccination." The greatest care was taken to have good virus, and everything necessary to insure success, as the account further states: "The persons attached to the expedition were several physicians with assistants, and 22 children who had not had the small pox, and were destined to preserve the valuable fluid by a successive vaccination from arm to arm on one after another in the course of the voyage."

After diffusing vaccination through Spanish America, and stablishing societies for its preservation, the Doctor determined to carry this "consolation of humanity," as he terms it, to Asia. A portion of the expedition was also sent to Peru, and it is said the vaccine disease was communicated to 50,000 persons without one unpleasant result. It is owing to the zeal and energetic labors of such men as Dr. Balmis that we have for a long time been comparatively free from those terrible epidemics of small pox that were once so common, and which threaten us again unless we carefully use the means to avert such calamities. The great evil is in the use of worthless or impure matter, as the following figures will prove:—

Of 558 cases of small pox 131 have been vaccinated once, 155 twice, 95 three times, 133 four times, and only 44 never. Of the 514 vaccinated, 285 have had the vaccine disease once, 103 twice, and 16 three times; 107 never. In 59 of the cases put down as having had the vaccine disease, it is evident that the matter employed was bad, as is shown by the sore or cicatrix on the arm, and from the fact that these men had unmodified small pox; thus making 166 cases in which vaccination was unsuccessful, either from worthless matter or carelessness in performing the operation. The virus furnished the Medical Department of the Army is very often worthless and harmful. Of 264 cases vaccinated only 147 were successful, and in these the failure I am confident, was not owing to the careless or ignorant manner of inserting the virus.

We are accountable for the failures of vaccination, for if good virus is used, and the operation performed by an expert, success will be the rule, and failure the exception. How then is vaccination to be made more effectual? First, it is evident that compulsory laws, bearing on the subject, must be enacted requiring children to be vaccinated at an early age, and no one should be considered protected unless pronounced so by some one authorized to vaccinate and competent to judge.

We must also devise means for obtaining and preserving genuine vaccine virus. We shall constantly meet with disappointments so long as we depend on that kept for sale in the shops, and by those who make it a business to traffic in the article. Let the medical societies in every city and county appoint as a board a certain number of their members, whose duty it shall be to always keep on hand, at whatever cost or labor, a perfectly reliable article of vaccine matter, from which source alone let physicians draw their supplies. In this way the traffic in this important article, and the impositions so often practiced by unprincipled and ignorant persons, will be at an end, and the blessings of vaccination generally enjoyed. Whatever errors or dereliction of duty there may be in regard to this matter in future, I trust there will be none that may be charged upon the medical profession.—*Am. Med. Times.*

